# On the crystallisation temperature of very high-density amorphous ice

**DOI:** 10.1039/c7cp08595h

**Published:** 2018-04-10

**Authors:** Josef N. Stern, Thomas Loerting

**Affiliations:** a Institute of Physical Chemistry , University of Innsbruck , A-6020 Innsbruck , Austria . Email: thomas.loerting@uibk.ac.at

## Abstract

The influence of the protocol of preparation on the crystallisation temperature *T*_*X*_ of very high-density amorphous ice (VHDA) was studied by varying the annealing pressure (1.1, 1.6 and 1.9 GPa) and temperature (160, 167 and 175 K, respectively). *T*_*X*_ increases by up to 4 K in the pressure range of 0.7 to 1.8 GPa for samples annealed at 1.9 GPa compared to samples annealed at 1.1 GPa. Concomitantly, secondary crystallisation channels are suppressed, indicating the absence of structural inhomogeneities. For VHDA prepared at 1.1 GPa and 1.6 GPa our results indicate such inhomogeneities, which we regard to be incompletely amorphized, distorted nanodomains of hexagonal ice that cannot be detected through X-ray diffraction experiments. VHDA prepared at high pressures and temperatures thus represents the amorphous state of water at >0.7 GPa least affected by nanocrystals that has been described so far. We expect the *T*_*X*_ obtained for the samples prepared in this manner to be close to the ultimate limit, *i.e.*, we do not consider it possible to raise the low-temperature border to the no-man's land notably further by changing the preparation protocol. An additional, considerable increase in this border will only be possible by working at much shorter time-scales, *e.g.*, by employing fast heating experiments.

## Abstract 2

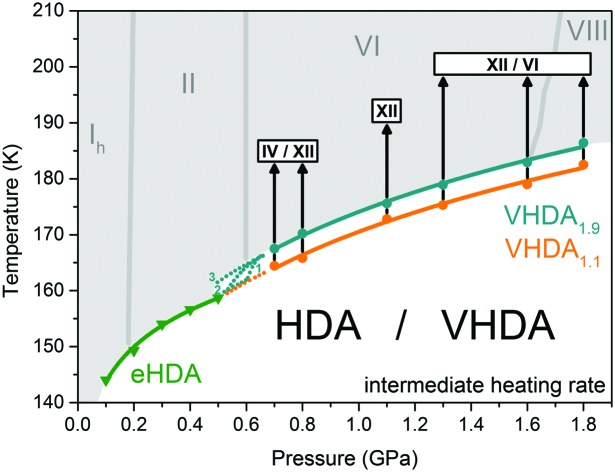
VHDA prepared at high pressures and temperatures appears to be mainly free of (nano)crystallinity. It is the thermally most stable amorphous solid phase of water at elevated pressures reported so far. Water's no man's land's low temperature boundary is thus shifted to higher temperatures by up to 4 K.

## Introduction

The discovery of a second amorphous solid phase of water with high density in 1984^1^ introduced a new facet to phase transitions. Besides the well-known concept of solid polymorphism^2^,^3^ – *i.e.*, the existence of more than one crystalline form for a substance – the concept of polyamorphism had to be coined for water. In fact, both the vast variety of crystalline phases and the existence of more than one amorphous solid form for water are considered to be among its numerous anomalies.^4^ Before high-density amorphous ice (HDA) was discovered, lower density glassy water had been known for almost 50 years as the phase condensing from gaseous water on a very cold substrate under vacuum.^5^,^6^ The relation between low-density amorphous ice (LDA) and HDA was heavily disputed in the past, especially concerning a possible reversible first-order phase transition between the two, questioning whether or not HDA can be considered a distinct amorphous glassy phase at all.^7^,^8^ To add to the complexity Loerting *et al.* described a third amorphous ice phase with even higher density than HDA in 2001 named very high-density amorphous ice, VHDA.^9^,^10^ VHDA can be prepared by annealing of HDA at elevated pressures *p* > 1 GPa or by pressurisation of LDA at elevated temperatures.^11^

HDA as originally prepared by pressure-induced amorphisation (PIA) of crystalline hexagonal ice I_h_ at low temperatures (∼77 K) by Mishima *et al.*^1^ results in a strained and unrelaxed phase and has later been referred to as “unannealed high-density amorphous ice” (uHDA).^12^ It is possible to relax and/or equilibrate this unrelaxed variant of HDA by isobaric annealing at low^13^ and higher pressures^12^ or by decompression of VHDA at elevated temperatures (140 K).^9^,^14^ HDA annealed at lower pressures *p* ≤ 0.2 GPa is of slightly lower density than uHDA and has thus been labelled and referred to as “expanded high-density amorphous ice” (eHDA).^12^,^15^ Both uHDA and eHDA are rather similar in terms of density as well as structure as demonstrated by cryoflotation and neutron diffraction experiments.^9^,^16^,^17^ However, they do differ substantially with respect to their state of relaxation and degree of homogeneity. Specifically, nano-scaled and distorted crystalline domains reminiscent of hexagonal ice were inferred to be the source of these inhomogeneities in the amorphous matrix by Tse *et al.*^18^–^20^ and Seidl *et al.*^21^ As a consequence of their nano-size they are elusive to structural characterisation such as XRD or neutron diffraction.^22^ One method to still identify their presence is to examine the amorphous phases’ thermal properties: at 1 bar HDA experiences a polyamorphic transition to LDA characterised by its transformation temperature *T*_poly_ and enthalpy Δ*H*_poly_. Compared to uHDA, *T*_poly_ is about 20 K higher^23^ for eHDA and Δ*H*_poly_ is reduced by up to 300 J mol^–1^.^24^ This evidently indicates that uHDA represents an unrelaxed, high-enthalpy state in contrast to eHDA. At elevated pressures *p* > 0.1 GPa the polyamorphic transition is avoided and HDA crystallises instead. In a study by Seidl *et al.* it was demonstrated that the crystallisation temperatures *T*_*X*_ for uHDA are considerably lower compared to those of eHDA at pressures 0.1–0.5 GPa.^21^ These findings have led to the conclusion that incomplete PIA of ice I_h_ at 77 K leaves behind distorted ice I_h_ within uHDA. In other words, a mechanical collapse as suggested by Tse and co-workers seems to be at the origin of the high-enthalpy nature of uHDA.^18^–^20^ These crystalline inhomogeneities then act as seeds which merely have to grow at the expense of the uHDA matrix. In relaxed eHDA seeds have to nucleate in the first place to then grow. *I.e.*, the activation energy to grow crystals from amorphous ice is decreased due to the presence of nanocrystalline domains. Similarly, the kinetics of crystal growth are accelerated in their presence, such that they lower *T*_*X*_ in comparison with the purely amorphous, homogeneous matrix. Furthermore, the nanocrystalline domains may allow for two competing crystallisation modes, where the first one is crystallisation of the purely amorphous material and the second one is growth of nanocrystallites. As a result, phase mixtures may crystallise from amorphous ices containing nanocrystals.

The study by Seidl *et al.* on HDA was extended by us to higher pressures *p* ≥ 0.7 GPa using VHDA.^25^ One of the primary aims was to examine whether the influence of (nano)crystalline remnants in uHDA on *T*_*X*_ can be observed also at elevated pressures. We demonstrated that this is the case up to 0.8 GPa, whereas at *p* ≥ 1.1 GPa uHDA and VHDA become indistinguishable in terms of *T*_*X*_ and crystallising phase mixtures. That is, at *p* ≥ 1.1 GPa structural inhomogeneities in uHDA seem to disappear upon its transition to VHDA. In particular, the influence of secondary crystallisation channels – *i.e.*, transformation to more than one crystalline phase of water – distinguishes VHDA from uHDA. uHDA does not even show traces of ice XII upon crystallisation at 0.7 GPa and 0.5 K min^–1^, while in VHDA ∼40% of the product yield is ice XII – the crystalline phase that predominantly crystallises at 0.8–1.6 GPa.^25^,^26^ In spite of these differences, the crystallisation studies of VHDA prepared at 1.1 GPa indicate that secondary crystallisation channels are not entirely suppressed. It is, thus, the aim of the present study to investigate whether by varying its preparation conditions secondary crystallisation channels in VHDA can indeed be suppressed completely. In other words, it is our intent to probe whether or not nanocrystalline remnants can be amorphised entirely by using higher pressures and temperatures for the preparation of VHDA. This would raise the low-temperature border to the no man's land – the area in the phase diagram of water where non-crystalline phases do not exist – even further than achieved in our recent work.^21^,^22^,^25^ We emphasise that in our study we attempt to shift the border to the no-man's land at a given, fixed time scale, *i.e.*, heating rate. By contrast, it is possible to shift the high-temperature border to the no-man's land to lower temperatures by working on shorter time scales^27^,^28^ or avoiding crystallisation completely^29^,^30^ by using fast or ultrafast droplet cooling techniques.

## Experimental

### Preparation of VHDA

The different VHDA samples were prepared from an initial state of uHDA, which was annealed at three different pressures (1.1, 1.6 and 1.9 GPa) to temperatures just prior to crystallisation (160, 167 and 175 K, respectively). To prevent friction the water was loaded into pre-cooled indium containers (with a weight of approximately 0.35 g).^21^ Force was applied in a uniaxial manner resulting in a semi-hydrostatic pressure distribution inside the sample. The resulting VHDA phases are labelled as VHDA_1.1_, VHDA_1.6_ and VHDA_1.9_ for clarity. We want to stress that the labels in this work serve the sole purpose of marking the pressure (and consequently temperature) at which VHDA was annealed.

VHDA samples were subsequently quenched with liquid nitrogen to 77 K and then brought to a given pressure of 0.7–1.8 GPa. At the target pressure the amorphous ice was heated isobarically with 0.5, 5 and 30 K min^–1^ at 0.7 and 0.8 GPa, and with 5 K min^–1^ at 1.1, 1.3, 1.6 and 1.8 GPa. Crystallisation was identified from the volume curves as a step-like volume change with temperature, marking the transformation of the amorphous to a crystalline phase/phase mixture, which was then characterised *ex situ* by powder XRD.

### Sample characterization: dilatometry, X-ray diffraction and calorimetry

Experiments were conducted by *in situ* volumetry using a piston cylinder setup in a material testing machine (ZWICK model BZ100/TL3S) and *ex situ* structural characterisation *via* powder X-ray diffraction (Siemens diffractometer, model D5000; Cu Kα_1_: 0.15418 nm, Cu Kα_2_ and Cu Kβ are filtered out) in *θ*–*θ* geometry as described in our previous study.^25^ We employ a Göbel-mirror for parallel beam optics. For calorimetric analysis a differential scanning calorimeter (Perkin Elmer, model DSC 8000) was used. Samples of approximately 10 mg were loaded into aluminium DSC crucibles. Volumetry, diffraction and calorimetry experiments were conducted in a precise and controlled thermal environment down to approximately liquid nitrogen temperature (77 K).

## Results

### uHDA → VHDA transformation

#### Dilatometry

**Fig. 1:**
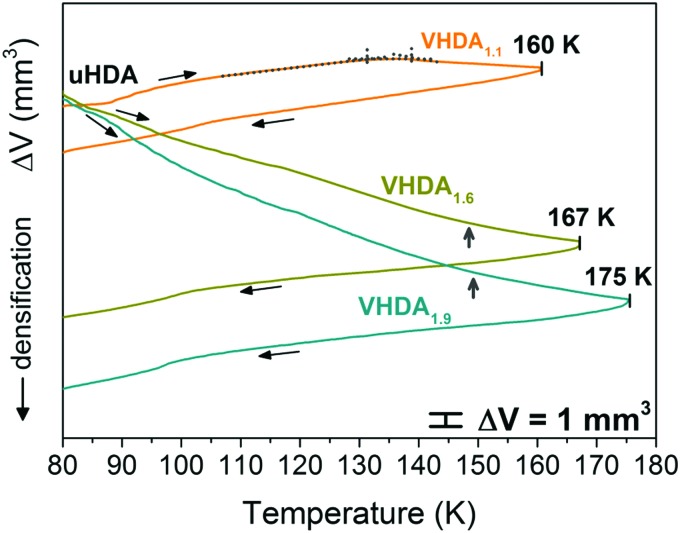
Volume curves for the preparation of very high-density amorphous ice at different pressures 1.1, 1.6 and 1.9 GPa. Amorphous ices are labelled by their pressure of preparation and colour coded orange (VHDA_1.1_), dark yellow (VHDA_1.6_) and cyan (VHDA_1.9_). Slope changes in the volume curves are marked with dotted grey lines in the case of VHDA_1.1_ and with vertical grey arrows in the case of VHDA_1.6_ and VHDA_1.9_.

When preparing VHDA samples at different pressures from an initial state of uHDA we observe a different evolution of density upon heating at each pressure as illustrated in Fig. 1. At 1.1 GPa the sample initially expands. This process of thermal expansion continues up to ∼130 K where it reaches a plateau, which is followed by densification above ∼140 K (marked for the orange curve of Fig. 1 with grey dotted lines). From there on the sample steadily densifies until the final temperature of 160 K is reached. The sign-change for the thermal expansion coefficient marks the uHDA → VHDA transition. This interpretation is backed by previous Raman spectroscopy data, where at 1.17 GPa a step-like transition of the coupled OH stretching band by approximately 35 cm^–1^ was observed at *T* ≈ 130 K indicating the polyamorphic uHDA → VHDA transformation.^31^

We have recently demonstrated that this onset in the change of slope shifts to lower temperatures when going to higher pressures (volume curves for uHDA in Fig. 3 in ref. 25). For 1.6 and 1.9 GPa uHDA shows densification in the whole temperature range up to 167 K and 175 K, respectively (see Fig. 1). That is, the change in slope has already shifted to <80 K, or in other words they are unannealed VHDA samples to begin with at *p* ≥ 1.6 GPa and 77 K. A slight flattening of the curves at 1.6 and 1.9 GPa can be noted however, starting at *T* > 145 K in both cases (marked in Fig. 1 with grey vertical arrows). We infer this to originate from the glass transition of the amorphous solid to the ultraviscous liquid. This is in accordance with previous dielectric spectroscopy results showing dielectric relaxation types typical of ultraviscous liquids at temperatures *T* ⪆ 140 K and pressures *p* ⪆ 1 GPa.^32^ However, since not only reversible effects but also the irreversible densification contribute to the shape of the volume curve, flattening cannot be directly assigned to the volumetric glass-to-liquid transition temperature.^33^

#### Powder X-ray diffraction

**Fig. 2:**
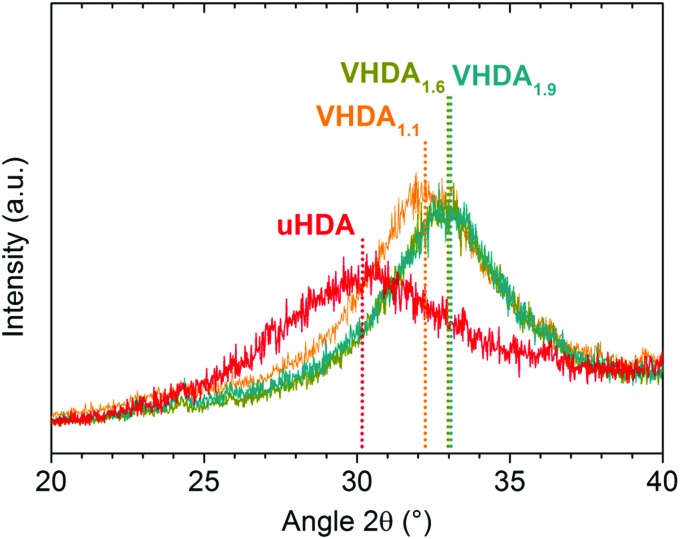
Diffractograms of unannealed high-density amorphous ice (in red) and differently prepared very high-density amorphous ices in orange (VHDA_1.1_), dark yellow (VHDA_1.6_) and cyan (VHDA_1.9_).

The powder X-ray diffractograms of VHDA produced under varying conditions and recovered at 77 K to atmospheric pressure (Fig. 2) confirm our interpretation of the dilatometry data, as shown in Fig. 1. Annealing VHDA at higher pressures to higher temperatures results in higher densities. This is reflected in the shift of the broad amorphous halo peak position (2*θ*) in Fig. 2. The diffractograms demonstrate that going from VHDA_1.1_ to VHDA_1.6_ and VHDA_1.9_ the halo peak shifts from ∼32.4° to ∼32.9° and ∼33.0° (see Table 1). For comparison, the uHDA halo peak is located at ∼30.5° as has been described in the literature.^14^,^22^ Densities were determined by means of cryoflotation,^16^*i.e.*, by varying a liquid N_2_/Ar mixture until the respective VHDA samples were suspended (Table 1).

**Table 1:** Peak positions 2*θ* (°) and full width at half maximum (FWHM) of the amorphous halo peaks, as well as densities *ρ* (g cm^–3^) as determined by buoyancy (cryoflotation^16^)

	Peak position 2*θ* (°)	FWHM	*ρ* (g cm^–3^)
VHDA_1.1_	32.4 ± 0.2	5.2 ± 0.2	1.25 ± 0.01^9^
VHDA_1.6_	32.8 ± 0.2	4.9 ± 0.5	1.26 ± 0.01
VHDA_1.9_	33.0 ± 0.2	4.8 ± 0.5	1.26 ± 0.01
uHDA	30.2 ± 0.2	7.7 ± 0.4	1.15 ± 0.01^9^

In addition to the peak position also the peak width changes significantly. The uHDA halo peak is much broader than all the VHDA halo peaks (see Table 1). Assuming that the broadening reflects structural inhomogeneities^34^ we note a trend of decreasing inhomogeneity upon increasing the preparation pressure for VHDA (see full-width at half maximum (FWHM) listed in Table 1).

#### Differential scanning calorimetry

**Fig. 3:**
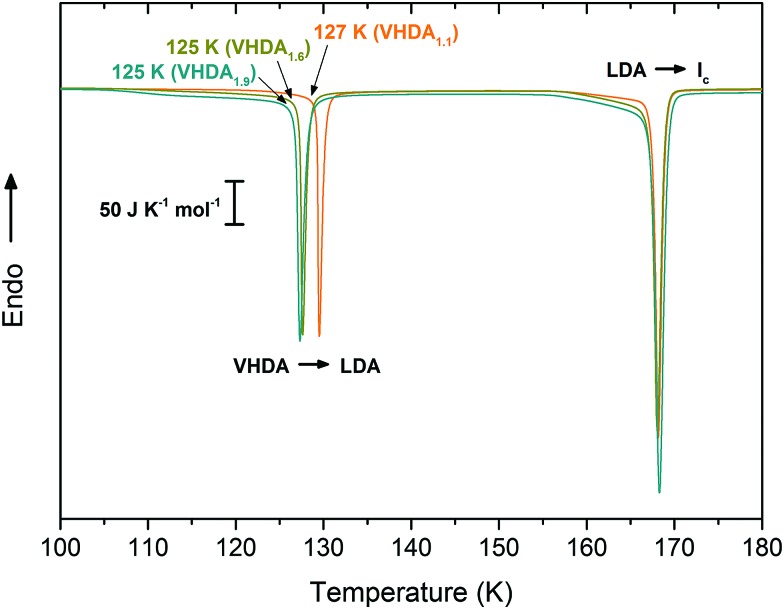
Differential scanning calorigrams of VHDA_1.1_, VHDA_1.6_ and VHDA_1.9_ recorded at atmospheric pressure and employing a heating rate of 10 K min^–1^ (VHDA_1.1_ in orange, VHDA_1.6_ in dark yellow and VHDA_1.9_ in cyan).

The VHDA samples were additionally studied at atmospheric pressure by differential scanning calorimetry. In Fig. 3, the first sharp exotherm marks the polyamorphic transition VHDA → LDA at *T*_poly_, and the second, larger exotherm marks LDA crystallisation to cubic ice I_c_ at *T*_*X*_. VHDA_1.1_ transforms at the highest (*T*_poly_ ∼ 127 K) and VHDA_1.9_ at the lowest temperature (*T*_poly_ ∼ 125 K) to LDA. This significant difference of 2 K indicates the denser, and hence more instable, nature of VHDA_1.9_ at 1 bar, which results in its lower thermal stability. By contrast *T*_*X*_ does not differ between the three variants of VHDA since the LDA formed after the polyamorphic transition represents the same metastable equilibrium state at 1 bar for all three variants.

### Crystallisation of VHDA at 0.7 and 0.8 GPa

**Fig. 4:**
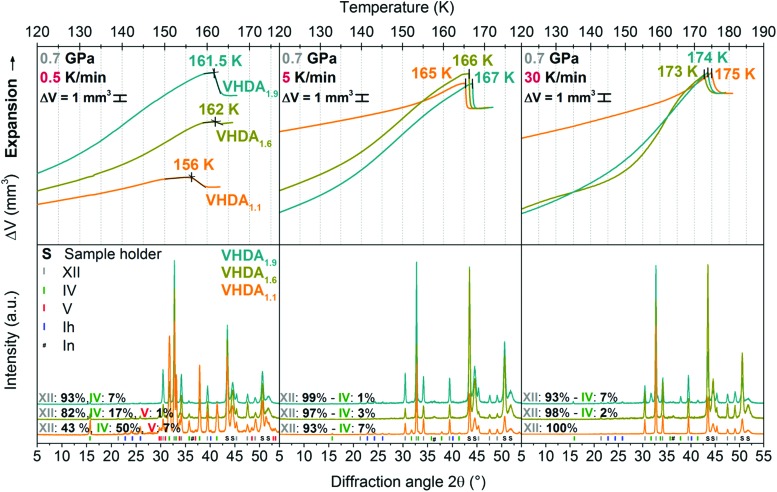
Upper panel: *In situ* volume curves of isobaric heating runs for VHDA_1.1_ (orange), VHDA_1.6_ (dark yellow) and VHDA_1.9_ (cyan) at 0.7 GPa with 0.5, 5 and 30 K min^–1^ heating rate. Lower panel: Structural characterisation by *ex situ* powder X-ray diffraction of the quench-recovered samples, colour coded accordingly. Diffractograms are stacked for clarity and labelled with the composition of their crystalline phase mixtures.

**Fig. 5:**
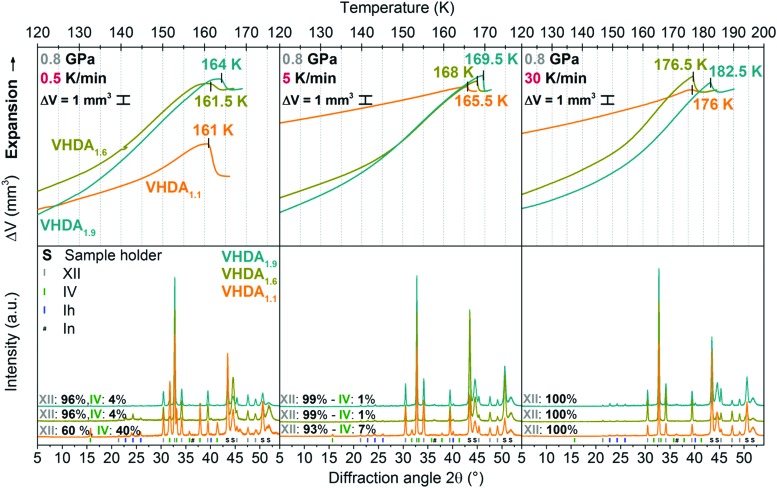
Same as Fig. 4, but for experiments at a pressure of 0.8 GPa.

To gain further insight into the nature of these differently prepared forms of VHDA their crystallisation upon isobaric heating was studied at elevated pressures using several heating rates. Fig. 4 and 5 exhibit results from individual isobaric heating experiments at 0.7 and 0.8 GPa for VHDA_1.1_, VHDA_1.6_ and VHDA_1.9_ at heating rates of 0.5, 5 and 30 K min^–1^. As has been described in the literature, transformation behaviour of the amorphous ices to crystalline phases upon isobaric heating changes both with pressure and heating rate.^21^,^22^,^25^,^26^,^31^ From the experiment we extract the onset crystallisation temperatures and composition of the crystallised product.

#### Crystallisation temperature *T*_*X*_

Upon heating VHDA the density decreases with temperature (Fig. 4 and 5). Previous studies have demonstrated that the evolution of the amorphous ices’ density prior to crystallisation is related to thermal expansion and reversible as well as irreversible structural relaxation processes.^33^ At lower pressures 0.7 and 0.8 GPa very high-density amorphous ices prepared at higher pressures (*i.e.*, VHDA_1.6_ and VHDA_1.9_) are rather far away from their respective thermodynamic equilibrium. That is why structural relaxation processes contribute more to the decrease of density with temperature in the cases of VHDA_1.6_ and VHDA_1.9_ than in the case of VHDA_1.1_ (the upper panels in Fig. 4 and 5). The density decrease with temperature continues for all variants until the limit of thermal stability is reached, where sudden densification occurs due to crystallisation (see rather sharp step in Fig. 4 and 5).

This indicates that the crystalline phase to which VHDA converts is of higher density (mainly ice XII, some ice IV; and sometimes traces of ice V). Similar to previous work,^25^*T*_*X*_ is defined by the intersection of two straight lines through linear parts in the volume curve just prior to and after the onset point of crystallisation (left upper panel, Fig. 4). The volume curves of Fig. 4 and 5 (upper panels) are aligned at the end point of crystallisation in cases of equal or very similar crystalline phase composition (see, *e.g.*, Fig. 4 upper panel, middle and right).

The crystalline phase composition is obtained from the intensity ratio of the most intense Bragg peaks by *ex situ* powder XRD characterisation (at ∼80 K and *p* ∼ 10^–1^ mbar, bottom panels in Fig. 4 and 5).^19^,^35^–^37^ Regarding *T*_*X*_ and taking a look into the differently prepared VHDA phases separately, trends are observed similar to the ones reported in the literature.^21^,^22^,^25^,^26^ That is, for a given amorphous phase and at a selected pressure *T*_*X*_ increases with heating rate. For instance, *T*_*X*_ for VHDA_1.6_ at 0.7 GPa changes from 162 K to 166 K and 173 K when going from 0.5 to 5 and 30 K min^–1^ heating rate. Also, when employing the same heating rate *T*_*X*_ increases with pressure, *e.g.*, VHDA_1.6_ from 167 to 169 K at 5 K min^–1^. The same can be observed for VHDA_1.1_ and VHDA_1.9_. Furthermore, *T*_*X*_ increases going from VHDA_1.1_ to VHDA_1.6_ and VHDA_1.9_ under identical experimental conditions, *i.e.*, heating with the same rate at the same pressure. VHDA_1.9_ crystallises at temperatures at least 2 K (and up to 6.5 K) above those of VHDA_1.1_ with the exception at 0.7 GPa/30 K min^–1^.

#### Phase composition

The products are composed of typically two crystalline phases (Table 2), indicating parallel crystallisation. This transformation process to more than one crystalline phase has been described in the literature for HDA at low pressures (0.1–0.5 GPa) and for HDA and VHDA at intermediate pressures (*p* > 0.7 GPa).^21^,^22^,^25^,^26^,^38^,^39^ These studies have shown that the relative yields of given crystalline phases at a specific pressure can be influenced by variation of the heating rate, as for each single phase different transformation kinetics are involved.^25^,^26^,^38^,^39^ At 0.7 and 0.8 GPa VHDA transforms to mainly ices XII and IV. In fact, when employing heating rates of 5 and 30 K min^–1^ ice XII is formed almost exclusively for VHDA_1.1_, VHDA_1.6_ and VHDA_1.9_. Only at a low heating rate (0.5 K min^–1^) a significant difference becomes notable: going from VHDA_1.1_ to VHDA_1.9_ the effect of parallel crystallisation decreases. Ice IV develops from a main crystallisation product in the case of VHDA_1.1_ (40–50%) to a by-phase (4–7%) for VHDA_1.9_. That is, secondary crystallisation channels are mostly suppressed for VHDA_1.9_.

**Table 2:** Phase mixtures from VHDA crystallisation experiments upon isobaric heating with 0.5, 5 and 30 K min^–1^; crystalline products were characterised *ex situ* by powder X-ray diffractometry at ∼80 K and 3 × 10^–1^ mbar

	VHDA_1.1_	VHDA_1.6_	VHDA_1.9_
	0.5 K min^–1^		
	V/IV/XII (%)	V/IV/XII (%)	V/IV/XII (%)
0.7 GPa	7/50/43	—/19/81	—/7/93
0.8 GPa	—/40/60	—/4/96	—/4/96

	VHDA_1.1_	VHDA_1.6_	VHDA_1.9_
	5 K min^–1^		
	IV/XII/VI (%)	IV/XII/VI (%)	IV/XII/VI (%)
0.7 GPa	7/93/—	3/97/—	1/99/—
0.8 GPa	7/93/—	1/99/—	1/99/—
1.1 GPa	3/97/—	—/100/—	—/97/3
1.3 GPa	2/97/1	—/85/15	—/91/9
1.6 GPa	5/94/<1	—/85/15	—/59/41
1.8 GPa	<1/48/51	—/20/80	—/2/98

	VHDA_1.1_	VHDA_1.6_	VHDA_1.9_
	30 K min^–1^		
	IV/XII (%)	IV/XII (%)	IV/XII (%)
0.7 GPa	—/100	2/98	7/93
0.8 GPa	—/100	<1/>99	<1/>99

### Crystallisation of VHDA at 1.1 to 1.8 GPa

**Fig. 6:**
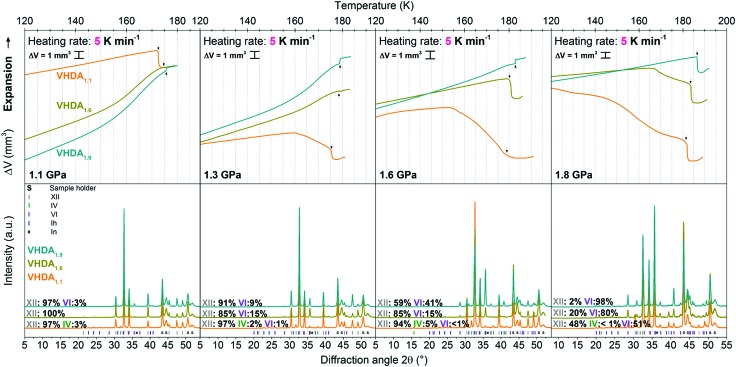
Upper panels *in situ* volume curves of isobaric heating runs for VHDA_1.1_ (orange), VHDA_1.6_ (dark yellow) and VHDA_1.9_ (cyan) in the pressure range 1.1–1.8 GPa with a heating rate of 5 K min^–1^ exclusively. Lower panels results of the corresponding structural characterisation by *ex situ* powder X-ray diffractometry of the quench recovered samples, colour coded correspondingly. Diffractograms are stacked for clarity and labelled with the composition of their crystalline phase mixtures.

**Fig. 7:**
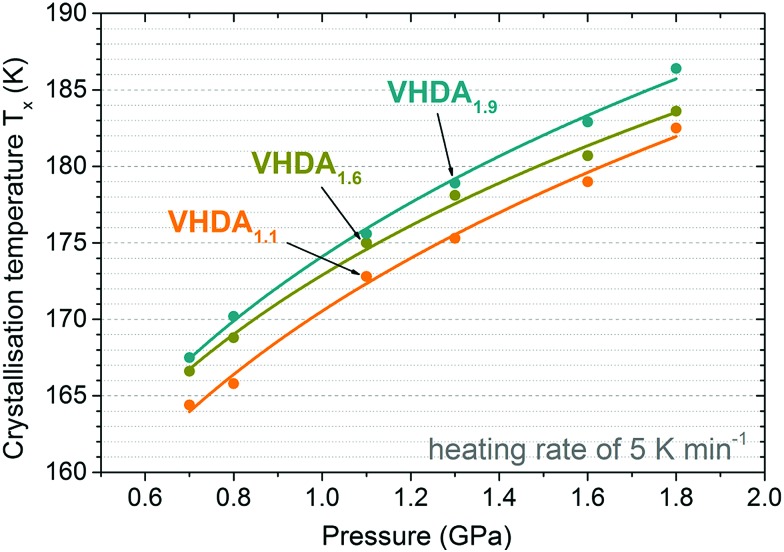
Crystallisation temperatures for differently prepared VHDAs at pressures 0.7–1.8 GPa, heated isobarically with a rate of 5 K min^–1^. The results of the differently prepared amorphous phases are colour coded accordingly (VHDA_1.1_ in orange VHDA_1.6_ in dark yellow and VHDA_1.9_ in cyan). Standard deviation is 0.2 K. Error bars are, thus, smaller than the symbol size.

The difference in crystallisation temperature Δ*T*_*X*_ for differently prepared VHDA prevails also at higher pressures up to 1.8 GPa (Fig. 6 and 7). In between VHDA_1.1_ and VHDA_1.9_ Δ*T*_*X*_ is approximately 4 K throughout the whole pressure range, employing a heating rate of 5 K min^–1^ (see Fig. 7). The evolution of the volume curves displayed in Fig. 6 is a reflection of the amorphous samples’ and the crystalline products’ varying densities. While VHDA_1.1_ apparently transforms to a denser phase upon crystallisation in all cases, VHDA_1.9_ in fact expands at pressures *p* < 1.8 GPa when crystallising. Bearing in mind that both VHDA_1.1_ and VHDA_1.9_ at pressures *p* < 1.6 GPa crystallise predominantly to ice XII of a fixed density, this implies that the densities of the amorphous matrices just before *T*_*X*_ are different. That is, the amorphous matrix has not reached an equilibrium density just before *T*_*X*_ at 1.1–1.3 GPa. At 1.8 GPa all three VHDA variants densify upon crystallisation, and in all cases the denser ice VI is now the crystalline phase primarily formed. The densification that VHDA_1.1_ (top panel, Fig. 6, 1.3–1.8 GPa) and VHDA_1.6_ (top panel, Fig. 6, 1.8 GPa) exhibit in a broad temperature range prior to the step-like transformation is due to relaxation of the amorphous matrix to a more dense one at pressures above their respective pressure of formation (1.1 GPa for VHDA_1.1_ and 1.6 GPa for VHDA_1.6_, respectively).

As demonstrated in Fig. 7 the same trend as at lower pressures (0.7 and 0.8 GPa) is observed at higher pressures 1.1–1.8 GPa regarding *T*_*X*_. That is, at the same pressure and heating rate *T*_*X*_ increases with pressure (and temperature) of VHDA preparation: *T*_*X*_ (VHDA_1.1_) < *T*_*X*_ (VHDA_1.6_) < *T*_*X*_ (VHDA_1.9_).

The reproducibility of the experimental results is rather high. Crystallisation temperatures from individual runs for the same type of experiment (*i.e.*, isobaric heating of the same VHDA phase at the same pressure and with the same heating rate) lie within 2 K, and the standard deviation as determined from several sets of identical experiments is approximately 0.2 K.

## Discussion

### Polyamorphic transitions at atmospheric pressure

As has been demonstrated for HDA in the literature the transformation to LDA at atmospheric pressure is much dependent on the amorphous ices’ state of relaxation.^23^,^40^ A transformation temperature of ∼116 K for uHDA, *e.g.*, is 20 K below that found for eHDA when being heated with 10 K min^–1^ at *p*_atm_.^40^ This reflects the unrelaxed, high-enthalpy nature of uHDA and can directly be associated with the presence of nanocrystalline domains contained in uHDA. Since the densities of uHDA and eHDA are very similar one would expect them to be roughly equal in terms of enthalpy. The difference in enthalpy, thus, requires an explanation that is not based on density – the presence of nanocrystallites provides such an explanation. By contrast, VHDA_1.1_ reproducibly transforms to LDA at temperatures ∼2 K higher than VHDA_1.6_ and VHDA_1.9_. This indicates that VHDA_1.9_ represents a high-enthalpy state at ambient pressure as well. However, because the density for VHDA_1.9_ is higher than the density for VHDA_1.1_ it is not possible to judge from these measurements on the presence or absence of nanocrystalline domains embedded in the amorphous matrix. To answer this question one needs to resort to measurements of the VHDA crystallisation kinetics.

### Suppression of type 1 crystallisation kinetics in VHDA_1.9_

Going from the lowest (0.7 GPa) to highest (1.8 GPa) pressure, the shift to ice VI as the crystalline phase predominantly formed upon crystallisation at the expense of ice XII can be observed for all differently prepared VHDA phases and is summarised in Table 2. The process of parallel crystallisation and its dependence on heating rate, but also pressure is well known for the amorphous ices. Salzmann *et al.* described this phenomenon in the case of HDA (uHDA) as a competition of transformation processes to different crystalline phases, being connected to different respective crystallisation kinetics.^26^ At a given pressure, that is depending on the heating rate, the relative yield of one crystalline phase in a mixture of crystalline phases can be influenced by choosing a low or a high heating rate. In this context Salzmann *et al.* labelled the processes with “type 1 kinetic behaviour” for transformation processes with slower kinetics starting at lower temperatures and “type 2 kinetic behaviour” for transformation processes related to faster kinetics starting at higher temperatures. This has also been observed in the case of VHDA (VHDA_1.1_).^25^ In fact, at lower pressures, *e.g.*, 0.7 GPa the relative yield of ice XII in the phase mixture may change from ∼40% to ∼90% and ultimately 100% when increasing the heating rate from 0.5 to 5 and 30 K min^–1^ (orange diffractograms in the lower panel of Fig. 4). Here, ice XII forms favourably and in greater amounts at higher heating rates and thus apparently transforms in a process connected to faster type 2 kinetics. Ice IV (and ice V at 0.5 K min^–1^ heating rate) consequently transforms with slower type 1 kinetics, and its formation is entirely suppressed when heating with a high rate of 30 K min^–1^. Also at 0.8 GPa the suppression of ice IV formation with increasing heating rate is observable (orange diffractograms in the lower panels of Fig. 5). While at a slow heating rate of 0.5 K min^–1^ ice IV is still formed in considerable amounts (∼40%), at higher heating rates of 5 and 30 K min^–1^ its relative yield decreases to 5–10% and ultimately diminishes to zero.

As has been demonstrated on the example of HDA^26^ the competition and interplay between transformation processes with different kinetics is not only dependent on heating rate, but also on pressure. That is, crystalline phases transforming with faster type 2 kinetics at lower pressures change their behaviour to slower type 1 kinetics when going to higher pressures with respect to other, denser crystalline phases – the formation of which is more favourable under these conditions and can now be considered of faster type 2. In the case of VHDA (VHDA_1.1_) it has been shown in the literature that the pronounced formation of ice IV when heating with low rates (0.5 K min^–1^) and at lower pressures (0.7, 0.8 GPa) decreases when going to higher pressures (Fig. 2 in ref. 25). The relation ice IV/type 1 kinetics – ice XII/type 2 kinetics is generally observed for VHDA_1.1_ in the whole pressure range 0.7–1.8 GPa and at all heating rates. However, at pressures *p* ≥ 1.3 GPa the formation of denser ice VI becomes increasingly favourable as is demonstrated in Table 2. And at 1.8 GPa the amount of ice VI formed upon crystallisation is already ∼50% when heating with a rate of 5 K min^–1^. In the frame of parallel transformations with different kinetic processes the formation of ice VI can now be considered of faster type 2, while crystallisation of ice IV is still of the slower type 1. Ice XII, however, must kinetically be related to a process intermediate with respect to ices IV and VI. This tendency is even more pronounced in the cases of VHDA_1.6_ and VHDA_1.9_. Already at 1.3 GPa considerable amounts (∼10% or more) in the crystalline phase mixtures are ice VI, and the formation of ice IV is entirely suppressed. And at 1.8 GPa the relative yield of ice VI after crystallisation from VHDA_1.9_ is almost 100%.

### Influence of annealing pressure and temperature on nanocrystals embedded in VHDA

The fact that the density of the examined amorphous phases is dependent on the pressure of preparation and is related to the broad halo peak in an X-ray diffractogram is well known in the literature.^9^,^14^,^41^ A shift to higher angles (2*θ*) as demonstrated in Fig. 2 is thus a reflection of an increase in density. The higher the pressure (and temperature) of preparation and thus the density of VHDA, the higher the temperatures at which it crystallises upon isobaric heating. In comparison with VHDA annealed at 1.1 GPa (to 160 K), the resulting amorphous phases of very high density are thermally more stable against transformation by up to approximately 4–5 K (in the case of VHDA_1.9_) throughout the whole pressure range of 0.7–1.8 GPa. Furthermore, VHDA_1.9_ shows a complete suppression of secondary crystallisation channels at the lowest (0.7 GPa) as well as the highest (1.8 GPa) examined pressure. Regarding ice XII, the shift from formation with faster type 2 kinetics (ice IV being the slower type 1 kinetics co-phase at *p* ≤ 0.8 GPa) to slower type 1 kinetics (ice VI being the faster type 2 kinetics co-phase at *p* ≥ 1.1 GPa) occurs at considerably lower pressures than is the case with VHDA_1.1_. At 1.8 GPa the relative yield of ice VI in the product's phase mixture for VHDA_1.9_ is already close to 100%, whereas in the case of VHDA_1.1_ it is ∼50% with a mixture containing ices VI, XII and even IV. The suppression of a secondary crystallisation channel even at low heating rates as well as increased transformation temperatures (*T*_*X*_ (VHDA_1.1_) < *T*_*X*_ (VHDA_1.6_) < *T*_*X*_ (VHDA_1.9_)) well demonstrates that the protocol of preparation for VHDA defines its homogeneity. In this context the term ‘homogeneity’ is related to the absence of crystalline remnants. As has been described in the literature these crystallites likely survive the pressure-induced amorphisation of hexagonal ice I_h_ when forming uHDA and are on the nano-scale, elusive to structural characterisation such as X-ray and neutron diffraction.^21^,^22^ When uHDA is annealed under pressure the nanocrystallites dissolve at least partially in the amorphous matrix prior to crystallisation. The higher the pressure of annealing the further away the system is from equilibrium conditions of the crystalline remnants. That is, the less likely the crystallites are to survive the annealing process when forming VHDA from uHDA. Or in other words, the higher the pressure and temperature HDA is annealed at, the more homogeneous the resulting VHDA.

### From higher to lower pressures: connecting the low-temperature boundary of no man's land

**Fig. 8:**
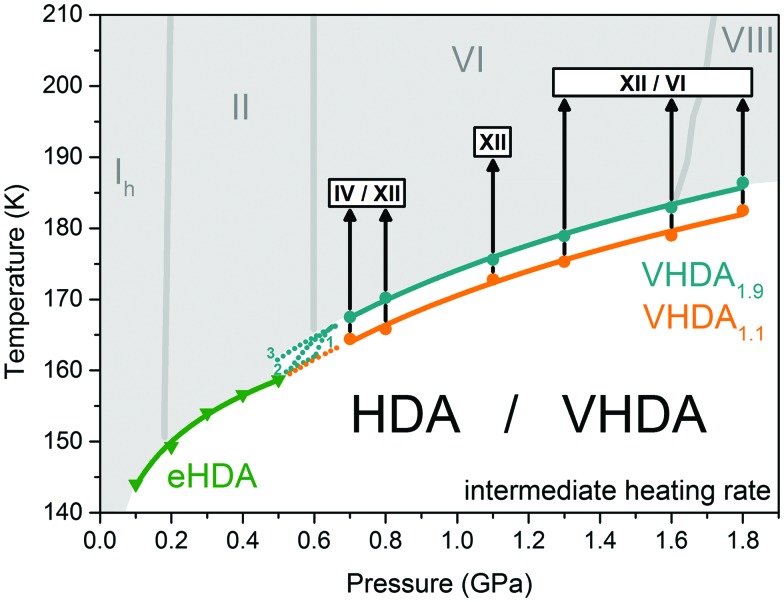
Crystallisation temperatures of eHDA (green) at *p* ≤ 0.5 GPa from Seidl *et al.*^21^ and differently prepared VHDA (VHDA_1.1_, orange and VHDA_1.9_, cyan) at *p* ≥ 0.7 GPa in the intermediate pressure regime. Dotted lines indicate possible scenarios connecting the crystallisation lines for eHDA and VHDA. Stable crystalline ice phases from the phase-diagram are marked in grey. Polymorphs (stable as well as metastable) crystallising from VHDA upon isobaric heating (black arrows) are marked by white boxes.

The results of our previous work on this topic^25^ indicated a rather smooth transition between the crystallisation lines of VHDA (VHDA_1.1_) and HDA (eHDA) applying intermediate heating rates (2 and 5 K min^–1^). The new outcomes for the more homogeneous and thermally stable VHDA_1.9_, however, draw a more complex picture (Fig. 8). The fact that the crystallisation temperatures of VHDA_1.9_ are consistently at least 4 K above those determined previously for VHDA_1.1_ allows for three scenarios, indicated by the numbered, dotted cyan lines in Fig. 8. In the first one the crystallisation lines connect discontinuously, marked by a pronounced kink in the boundary to the no man's land. Although such a kink would not be direct evidence, indirectly it might indicate a first-order transition between HDA and VHDA. Such a first-order relation has been described previously in isothermal compression experiments, following the polyamorphic transition path of LDA → HDA → VHDA at 125 K.^11^ However, it would require time scales to be slow enough for interconversion between HDA and VHDA prior to crystallisation for the considered heating rates (*i.e.*, intermediate rates of 2 or 5 K min^–1^, respectively). In a second scenario the crystallisation lines may merge continuously, pointing towards a higher order transition. Also in this scenario time scales would have to allow for polyamorphic transitions prior to crystallisation. The third possible scenario would not see a conjunction of crystallisation lines at all at elevated pressures. Here, *T*_*X*_ (HDA) and *T*_*X*_ (VHDA) would intersect at much lower pressures.

A further interesting Gedanken experiment is the formation of eHDA *via* VHDA_1.9_ instead of the previously employed route *via* VHDA_1.1_. That is, preparing eHDA by decompressing VHDA_1.9_ at elevated temperatures (140 K) to lower pressures, *e.g.*, 0.2 GPa. Comparing the crystallisation behaviour of such differently prepared eHDA variants might yield an answer to the question of whether eHDA prepared *via* VHDA_1.1_ is a homogeneous amorphous phase or if the inhomogeneities apparently prevalent in VHDA_1.1_ ‘survive’ the elevated-temperature decompression to eHDA. In the former case, eHDA crystallisation temperatures would be indistinguishable, no matter whether VHDA_1.9_ or VHDA_1.1_ is employed for eHDA formation. In the latter case we would expect eHDA prepared *via* the homogeneous VHDA_1.9_ to be thermally more stable against crystallisation than eHDA prepared *via* less homogeneous VHDA_1.1_. This Gedanken experiment will be realized in future studies.

## Summary and outlook

The influence of annealing pressure and temperature when preparing VHDA from uHDA on the crystallisation behaviour of VHDA upon isobaric heating at elevated pressures 0.7–1.8 GPa was studied regarding its dependence on heating rate and pressure. The amorphous phases of very high density are indexed with the pressure at which they were annealed (VHDA_1.1_, VHDA_1.6_ and VHDA_1.9_). It could be shown that the higher the annealing pressure and consequently temperature, the higher the thermal stability against crystallisation at all examined pressures. Crystallisation temperatures of VHDA as prepared in the past (by annealing of uHDA at 1.1 GPa to 160 K^9^) were consistently ∼4 K below those of VHDA annealed at 1.9 GPa to 175 K. Furthermore, the process of parallel crystallisation is almost entirely suppressed at the lowest and highest examined pressures in the case of VHDA_1.9_. We, thus, consider VHDA prepared at high pressures and temperatures to be the most relaxed and homogeneous amorphous matrix of very high density described so far. In the context of the discussion about nanocrystalline domains embedded in the matrix of uHDA, this implies that the amorphisation remains incomplete after annealing at 1.1 GPa, whereas it comes much closer to completion at 1.9 GPa. In other words, we regard VHDA_1.9_ to be close to or fully fully amorphised, nearly or entirely free from distorted, crystalline nanodomains.

In the future it will be of interest to examine the transformation of the most stable amorphous phase of very high density (VHDA_1.9_) at pressures *p* < 0.7 GPa. In the authors’ previous study^25^ it was shown that the crystallisation temperatures of VHDA_1.1_ at pressures *p* ≥ 0.7 seem to merge perfectly with those obtained for eHDA at pressures *p* ≤ 0.5 GPa in the literature.^21^ The new results, however, indicate that the transformation of the most thermally stable form of very high density (VHDA_1.9_) occurs at notably higher temperatures. It would be rather interesting to see at which pressure the crystallisation temperatures of VHDA_1.9_ and eHDA do converge.

## Conflicts of interest

There are no conflicts to declare.
